# Invisible victims of border conflict: health and wellbeing of border communities between Cambodia and Thailand

**DOI:** 10.3389/ijph.2026.1609740

**Published:** 2026-06-10

**Authors:** Virak Sorn

**Affiliations:** Faculty of Health Sciences and Biotechnology, University of Puthisastra, Phnom Penh, Cambodia

**Keywords:** border conflicts, health impacts, humanitarian interventions, invisible victims, mental health

## Introduction

Border conflicts are often framed in geopolitical and security terms; however, their implications for civilian health and wellbeing remain insufficiently examined. The recent escalation of tensions along the Cambodia–Thailand border has created a significant humanitarian and public health challenge for affected communities. Between May 2025 and early 2026, intermittent armed confrontations led to widespread displacement, civilian casualties, and disruptions to essential services. Reports indicate that hundreds of thousands of individuals were displaced, while healthcare facilities, schools, and critical infrastructure were damaged or temporarily closed [1].

These tensions are rooted in longstanding territorial disputes, particularly around the Preah Vihear Temple, a UNESCO World Heritage site. Although international legal rulings have clarified sovereignty over the temple, ambiguities regarding surrounding areas have contributed to recurring disputes. Beyond geopolitical considerations, the Cambodia–Thailand border represents a highly interconnected socioeconomic space, where communities depend on cross-border mobility for healthcare, trade, and employment [1]. Consequently, even localized conflicts can have disproportionate effects on population health.

The health impacts of armed conflict extend beyond immediate injury and mortality. Conflict disrupts environmental and structural determinants of health, including access to clean water, food systems, and healthcare services [2]. Displacement and overcrowding increase the risk of communicable diseases, while disruptions to routine care limit access for vulnerable groups such as children, pregnant women, and individuals with chronic conditions [3]. At the same time, exposure to violence, uncertainty, and economic hardship contributes to significant psychological distress. Evidence from affected provinces in Cambodia indicates that nearly two-thirds of displaced families experience severe emotional stress following conflict-related disruptions [4].

## Conceptual framework

This commentary conceptualizes border conflict as a structural determinant of health operating through interconnected pathways. Political instability weakens governance and cross-border cooperation, disrupting health systems through reduced service delivery, damaged infrastructure, and fragmented disease surveillance. At the community level, conflict alters key social determinants—including income, mobility, food security, and living conditions—thereby increasing vulnerability to communicable diseases, untreated chronic conditions, and mental health disorders. As a result, border communities become “*invisible victims*” of geopolitical tensions, facing risks that extend beyond immediate physical harm. In an increasingly interconnected world, these dynamics underscore the need to view border conflict not only as a political issue but also as a public health crisis requiring coordinated responses.

## Public health consequences of border tensions

### Disruption of healthcare access

Healthcare access in border regions often depends on cross-border mobility, particularly in rural and underserved areas. Movement restrictions, heightened security, and border closures can prevent individuals from accessing essential services. Pregnant women, patients with chronic diseases, and those requiring emergency care are especially affected, often facing delays or limited access to specialized treatment [3, 5]. In such settings, cross-border healthcare functions as an informal but critical system; its disruption can significantly worsen health outcomes.

### Mental health and psychosocial stress

Living in areas affected by geopolitical tension creates an environment of persistent uncertainty and fear. Military presence, exposure to violence, and the threat of displacement contribute to heightened psychological distress among residents [4]. Prolonged exposure increases the risk of anxiety, depression, and trauma-related disorders. Children and adolescents are particularly vulnerable, especially when education is disrupted and households face economic instability [6].

### Livelihood and food security impacts

The livelihoods of numerous households in the Cambodia–Thailand borderland are intrinsically linked to agriculture, cross-border commerce, and informal labor. Consequently, border closures and military tensions severely disrupt local microeconomies by restricting commercial flow and employment. The resulting economic contraction directly diminishes household purchasing power, thereby compromising nutritional security and the utilization of healthcare services and overall wellbeing. Additionally, this financial instability often drives population displacement, creating compounding challenges for vulnerable groups seeking continuous medical care and social support networks [7].

### Risks of communicable diseases

The destabilization of public health infrastructure in border regions significantly amplifies the risk of infectious disease outbreaks. Geographically contiguous areas necessitate integrated surveillance and response protocols to effectively monitor and mitigate vector-borne diseases such as dengue and malaria, alongside other communicable pathogens [1]. However, political tensions that undermine cross-border cooperation often result in the fragmentation of these critical epidemiological networks. Furthermore, forced displacement and mass migration exacerbate these vulnerabilities by disrupting routine immunization schedules and impeding comprehensive disease prevention initiatives.

## Vulnerable populations in border communities

The adverse impacts of border tensions are inequitably distributed, critically affecting demographics that depend on consistent clinical care—such as pediatric and aging populations, pregnant women, and those individuals with chronic diseases. The wellbeing of these groups is inextricably linked to the continuous provision of accessible healthcare and socio-environmental stability. Furthermore, informal cross-border traders and migrant workers face acute precarity, as their livelihoods are entirely predicated on transnational mobility [8]. Consequently, border closures and movement restrictions precipitate a dual crisis for these demographics: severe income attrition coupled with diminished healthcare access, thereby significantly compounding their risk of adverse health outcomes.

## The importance of cross-border health cooperation

Public health paradigms in borderlands inherently transcend sovereign demarcations. The efficacy of epidemiological surveillance, acute emergency response, and continuous healthcare delivery is heavily contingent upon robust bilateral collaboration between the counties. Maintaining care continuity and public health resilience in the face of geopolitical conflict requires strengthening cross-border health diplomacy [9]. Furthermore, supranational entities, notably the World Health Organization (WHO) and the Association of Southeast Asian Nations (ASEAN), are instrumental in facilitating transnational health initiatives and mediating bilateral discourse to strengthen healthcare services among people in affected areas. Implementing cooperative frameworks that center on health security and humanitarian imperatives is critical for attenuating the adverse epidemiological consequences of regional disputes [10].

## Policy recommendations

Addressing the health impacts of border conflict requires comprehensive, multi-level policy responses that extend beyond short-term humanitarian interventions.

- First, governments should institutionalize cross-border health coordination frameworks to ensure continuity of care during periods of instability. This includes formal agreements for real-time data sharing, joint disease surveillance, and coordinated outbreak response. Interoperable health information systems and standardized reporting protocols can strengthen early detection, while formal referral mechanisms enable timely access to cross-border care.
- Second, expanding decentralized and digital healthcare delivery is essential to ensure equitable access among populations facing mobility constraints. Telemedicine, mobile health platforms, and community-based outreach can connect underserved populations with higher-level services, reducing disruptions during crises.
- Third, mental health and psychosocial support services should be integrated into primary healthcare systems. Conflict-affected populations face elevated risks of anxiety, depression, and trauma-related disorders. Community-based and trauma-informed approaches—including psychological first aid, peer support, and school-based interventions—are critical. Strengthening provider capacity is also necessary to improve access and reduce stigma.
- Finally, health diplomacy should be prioritized to sustain collaboration despite political tensions. Policymakers must integrate the needs of border communities into national health security strategies and regional frameworks. Leveraging regional platforms can facilitate dialogue, resource mobilization, and coordinated responses that prioritize humanitarian access and system resilience.

### Conclusion

The impacts of border disputes extend profoundly beyond sovereign and territorial demarcations, precipitating severe, localized public health crises. Communities residing in the Cambodia–Thailand borderlands endure systemic disruptions to healthcare access, economic viability, and psychosocial stability during geopolitical escalations. Recognizing these demographics as the “*invisible victims*” of conflict underscores the urgent necessity for enhanced bilateral health cooperation, targeted community-based interventions, and responsive policy frameworks. Ultimately, safeguarding the health of these border populations is simultaneously a fundamental humanitarian obligation and a critical pillar of broader regional health security.
